# Impact of GH and IGF-I excess on nervous and vascular retinal structure in newly diagnosed acromegaly patients

**DOI:** 10.1007/s11102-025-01565-6

**Published:** 2025-10-10

**Authors:** Rosa Pirchio, Renata S. Auriemma, Gilda Cennamo, Daniela Montorio, Domenico Solari, Luigi M. Cavallo, Rosario Pivonello, Annamaria Colao

**Affiliations:** 1 Dipartimento di Medicina Clinica e Chirurgia, Sezione di Endocrinologia, Università Federico II di Napoli, Naples, Italy; 2 Public Health Department, University of Naples “Federico II”, Naples, Italy; 3 Department of Neurosciences, Reproductive Sciences and Dentistry, University of Naples “Federico II”, Naples, Italy; 4 Unesco Chair for Health Education and Sustainable Development, “Federico II” University, Naples, Italy

**Keywords:** Acromegaly, GH, IGF-I, Retina, OCT, Vessel density

## Abstract

**Purpose:**

To investigate nervous and vasculare structure of retina in naïve acromegaly patients using Spectral Domain Optical Coherence Tomography (SD-OCT) and Optical Coherence Tomography Angiography (OCTA).

**Methods:**

Prospective case-control study. Twenty-four eyes of 12 naïve acromegaly patients (8 men, 4 women, mean age 49.1 ± 12.3 years) without chiasmal compression by pituitary adenoma and 24 eyes of 12 healthy controls were evaluated in this study. In both groups were performed SD-OCT, to assess ganglion cell complex (GCC) and retinal nerve fiber layers (RNFL) thickness, and OCTA, to assess the vessel density (VD) of the superficial capillary plexus (SCP), deep capillary plexus (DCP), radial peripapillary capillary (RPC) and choriocapillaris (CC). In patient group, OCT parameters were correlated to hormonal (GH, IGF-I) and metabolic (glucose and lipid profile) parameters.

**Results:**

GCC (*p* = 0.001) and RNFL average (*p* = 0.007) were significantly lower in patients than in controls. At the regression analysis, adenoma volume (*p* < 0.001) and diagnostic delay (*p* < 0.001) before diagnosis were the best predictor of GCC and RNFL average, respectively. VD in SCP (*p* < 0.001), DCP (*p* = 0.009), and RPC (*p* = 0.03) was significantly lower in patients as compared to controls. VD in RPC was significantly lower in patients with diagnostic delay above 8.5 years (median, *p* = 0.009) and fasting glucose above 90 mg/dl (median, *p* = 0.04) as compared to those below.

**Conclusion:**

As acromegaly patients exhibited an impairment either in nervous and in vascular retinal structure at diagnosis, even in the absence of chiasmal compression, SD-OCT and OCTA may represents potential tools to detect retinal damage in these patients.

## Introduction

Acromegaly is a slowly progressive disease resulting from the increased release of growth hormone (GH) and, consequently, *Insulin- like growth factor* 1 (IGF-I) induced in most cases by a GH-secreting pituitary adenoma [1]. GH and IGF-I receptors are widely expressed in human body, as a consequence the prolonged exposure to GH and IGF-I excess induces progressive somatic modifications and several systemic complications, mainly including cardiovascular, respiratory, and metabolic ones [2, 3]. Besides these well-known comorbidities, ophthalmic tissues and their functions have also been demonstrated to be influenced by acromegaly disease [4–7]. Indeed, GH receptors have been found in the cornea, choroid coat, and retina [8], whereas IGF-I receptors have been found in cultured retinal pigment epithelial cells [9–11]. Therefore, GH may affect either directly [7] and through IGF-I [9] eye development and function. Consistently, ophthalmic impairment has been reported both in patients with GH deficiency [12, 13] and excess [4–7]. Moreover, the optic nerve could be damaged by the protracted compression of optic chiasm due to the presence of a pituitary macroadenoma in patients with acromegaly, leading to retrograde axonal degeneration [14]. Nevertheless, the ophthalmic impact of GH excess needs to be elucidated. The availability of new tools able to evaluate changes in nervous and vascular structures of the retina such as optical coherence tomography (OCT) has provided promising results. Measurement of the circumpapillary retinal nerve fibre layer (RNFL) and the ganglion cell complex (GCC) thickness by spectral domain optical coherence tomography (SD-OCT) may provide an estimation of visual outcome, being a lower thickness associated to a worse visual outcome [14]. Some other recent studies have evaluated retinal and choroidal structures in acromegaly patients using SD-OCT, although with conflicting results [15–20]. Of note, all these studies evaluated patients after several years from the diagnosis, already treated for acromegaly with neurosurgery or somatostatin receptor ligands (SRLs) [12–17]. Further, pituitary macroadenomas have been reported to induce RNFL and GCC thinning even in the absence of chiasmal compression [21], suggesting that pituitary adenomas *per se* may cause retinal ganglion cell dysfunction even in the absence of a compressive effect on the chiasm. Moreover, a significantly lower average RNFL has been demonstrated in patients with GH-secreting tumors as compared to patients with other pituitary adenoma hystotypes [21], suggesting that additional mechanisms could be involved in retinal neuronal damage in the former group, besides the burden of tumor mass. However, no data are available to date in this respect.

OCT angiography (OCTA) is a non-invasive method developed for the reconstruction of the three-dimensional chorioretinal vascular structure, which is currently used for the diagnosis of optic neuropathies [22–24]. To date, few studies have been conducted using OCTA to evaluate a potential vascular damage in patients with acromegaly [25–28], reporting a decrease in vessel density in several macular areas in patients compared to controls. However, also these studies evaluated patients after several years from the diagnosis who were already treated for acromegaly, mainly with neurosurgery. This is an important limitation since a recovery of retinal damage after neurosurgical treatment has been demonstrated in both nervous and vascular structure (29).

To date, no study has specifically investigated the effects of acromegaly in treatment-naïve patients and whether among pituitary adenomas GH-secreting tumors not inducing optic chiasm compression might induce damage of retinal nervous and vascular structures due to a direct effect of GH and IGF-I excess is yet to be fully elucidated. Further, the role and burden of acromegaly comorbidities, mainly metabolic ones, on retinal damage has not been investigated. Therefore, the purpose of current prospective study was three steps, aiming at investigating: (1) retinal nerve thickness using SD-OCT and the retinal and choriocapillaris vascular networks in macular and in peripapillary regions using OCTA in patients with GH and IGF-I excess resulting in acromegaly disease due to a pituitary adenoma without chiasmal compression compared to healthy controls; (2) the potential role of SD-OCT and OCTA in early diagnosis of retinal impairment in acromegaly patients; and (3) the impact of acromegaly complications, particularly hypertension, hyperglycemia, and dyslipidemia, as additional factors of retinal structure damage.

## Patients and methods

### Inclusion and exclusion criteria

The current prospective case-control study included patients with a well-established diagnosis of acromegaly based on international diagnostic criteria [30]. These patients were evaluated at acromegaly diagnosis, prior to any acromegaly treatment. Main inclusion criteria were: (1) age > 18 years; (2) diagnosis of acromegaly; (3) evidence at pituitary magnetic resonance imaging (MRI) of a pituitary adenoma, without chiasm impingement. Exclusion criteria were: (1) any previous treatment for pituitary adenoma; (2) presence of congenital eye disorders; (3) myopia > 6 dioptres; (4) previous ophthalmic surgery; (5) previous diagnosis of glaucoma; (6) any optic disc anomaly; (7) significant lens opacities or any macular disease; (8) low-quality OCTA and SD-OCT images.

Among people referring to the Eye Clinic of University of Naples “Federico II” to perform an ophthalmic evaluation, those aged > 18 years matching the above exclusion criteria from point 2 to 7 were asked to participate in the study as control group. In these subjects, the diagnoses of diabetes mellitus and arterial hypertension were excluded according to the international guidelines [31, 32].

All control subjects provide the written informed consent to partecipate in this study and underwent to the same ophthalmic evaluation of acromegaly patients.

## Patients

A total of twenty-four eyes in twelve consecutive acromegalic patients attending the Neuroendocrine Disease Unit between 1 st January 2018 and 31 st July 2019 (8 men, 4 women, mean age 49.1 ± 12.3 years) were included in the current study. The diagnosis of acromegaly was defined based on the international guidelines [30].

At study entry, diagnostic delay lenght was evaluated based on the onset of patognomonic acromegaly symptoms and on somatic changes obtained through the evaluation of picture series for each patient. Medical history and comorbidities, such as arterial hypertension, impairment in glucose metabolism (glucose intolerance and/or overt diabetes mellitus), and dyslipidemia, were also recorded. MRI revealed a pituitary microadenoma in 1 patient (8.3%) and a macroadenoma in 11 (91.7%). Twenty-four eyes in twelve healthy subjects (5 men, 7 women, mean age 50.3 ± 11 years), age and gender-matched to patients, with a normal ophthalmic examination were included as a control group.

To find a significant difference between the two groups in primary endpoints by large effect size (Cohen’s d = 0.8), group sample sizes of 8 was found to achieve a 90% power to reject the null hypothesis of equal means with a significance level (alpha) of 0.1 using a two-sided two-sample equal-variance t-test. With an expected dropout rate of 20%, the sample size was increased to at least 10 patients for each group to be enrolled.

Patient profile at study entry is shown in Table 1.

**Table 1:** Patients’ profile at study entry

Age, (years)	49.1 ± 12.3
M/F,(n)	8/4
GH,(ng/ml)	15.8 ± 20.9
IGF-I,(ng/ml)	813.5 ± 258.4
Microadenoma,(n,%)	1 (8.3)
Macroadenoma,(n,%)	11 (91.7)
Tumor volume,(ml)	2162.3 ± 1930.3
Fasting glucose,(mg/dl)	95.3 ± 18
Glucose impairment,(n,%)	6 (50)
Diabetes mellitus,(n,%)	2 (16.7)
IFG,(n,%)	2 (16.7)
IGT,(n,%)	2 (16.7)
Hypertension,(n,%)	5 (41.7)
Hypercholesterolemia,(n,%)	5 (41.7)
Hypertriglyceridemia,(n,%)	4 (33.3)

## Study protocol

Both eyes of each subject were examined as acromegaly has been reported to potentially affect non-identically the eyes [33, 34]. In all acromegaly patients, hormonal (GH, IGF-I) and metabolic parameters (blood pressure, fasting glucose, fasting insulin, HbA1c, total cholesterol, triglycerides, HDL and LDL cholesterol) were evaluated at diagnosis. Metabolic and cardiovascular complications were assessed according to international guidelines [35].

As per internal protocol, as soon as the diagnosis of acromegaly was confirmed and before starting any medical or surgical treatment for acromegaly, each subject underwent a complete ophthalmic evaluation including: slit-lamp biomicroscopy, fundus examination with a + 90 D lens, automatic perimetry test (Humphrey Field Analyzer using the 30 − 2 SITA-Standard algorithm of the Humphrey perimeter; Carl Zeiss Meditec, Dublin, CA, USA), spectral domain-OCT (SD-OCT) and OCTA (software ReVue XR version 2017.1.0.151, Optovue Inc., Fremont, CA, USA).

Similarly to acromegaly patients, control subjects recruited from the Eye Clinic of University of Naples “Federico II” underwent the same ophthalmic evaluation, including automatic perimetry test, SD-OCT, and OCTA.

The study adhered to the tenets of the Declaration of Helsinki. All patients and controls provided a written informed consent with respect to the Italian privacy policy to participate in the study. The study was approved by the Institutional Review Board of the University of Naples “Federico II” (NCT04840771).

## Assays

In acromegaly patients, hormonal and biochemical parameters were measured by standard methods. The determination of serum hormones levels were obtained with the solid phase chemiluminescent immunometric method (IMMULITE 2000 hGH, Siemens, Erlangen, Germany) using calibrators recommended by the WHO (IS 98/574 for hGH and IS 87/518 for IGF-1). GH levels were determined using a chemiluminescent immunometric assay (Liaison XL Analyzer, Diasorin), calibrated to the WHO 98/574 IS. The lower detection limit is 0 µg/L, while analytical sensitivity is 0.095–0.1 µg/L. The intra-assay and interassay CVs are 1.93–4.73% and 3.76–6.25%, respectively. IGF-1 values were measured with a chemiluminescent immunometric assay (Liaison XL Analyzer, Diasorin), calibrated to the WHO 02/254 IS. The assay has a detection range from 0 to 1500 µg/L, and an analytical sensitivity of 3 µg/L. The intra-assay and inter-assay CVs are 3–5.1% and 5.6–9.6%, respectively.

## Pituitary imaging

In acromegaly patients, magnetic resonance imaging (MRI) was performed on 1.5 T scanners, using a T1-weighted gradient recalled-echo (repetition time 200–300 ms; echo-time 10–12 ms; flip angle 90) in sagittal and coronal planes. In each measurement, 7–10 slides were obtained with a slice thickness of 2–3 mm. The scans were repeated before and after the administration of 0.1 mmol DTPA-gadolinium chelate (diethylenetriamine pentacetate). The patients were placed in the same position in each exam to obtain comparable slices. Tumor volume was calculated in line with De Chiro and Nelson formula: volume = height x length x width x π/6.

### Visual field

Visual field perimetry (Humprey Field analyzer with Swedish interactive thresholding algorithm (SITA) standard 30–2test program (Carl Zeiss Meditec, Dublin, CA, USA) was considered reliable when fixation losses were less than 20%, and false-positive and false-negative errors were less than 15%. The perimeter software was used to calculate mean deviation (MD) and pattern standard deviation (PSD).

## Spectral domain optical coherence tomography

The mean circumpapillary retinal nerve fibre layer (RNFL) and ganglion cell complex (GCC) thickness were evaluated, after pupillary dilation (minimum diameter 5 mm), with SD-OCT (software ReVue XR version 2017.1.0.151, Optovue Inc., Fremont, CA, USA) which captures 26,000 axial scans (A-scans) per second and provides a 5-µm depth resolution in tissue. The optic nerve head map protocol was used to evaluate the circumpapillary RNFL. This protocol generates a circumpapillary RNFL thickness map based on measurements obtained around a circle 3.45 mm in diameter centred on the optic disc. The GCC scan was centred 1-mm temporal to the fovea and covered a square grid (7 mm × 7 mm) on the central macula, and GCC thickness was measured from the internal limiting membrane to the outer boundary of the inner plexiform layer [36].

Only high-quality images, as defined by a signal strength index above 40, were accepted. Two experienced ophthalmologists (GC, DM), blinded to patient’s data, performed SD-OCT evaluations. The examiners rejected scans that had motion artefacts, poor centration, incorrect segmentation or poor focus.

## OCT angiography

OCTA images with the Optovue Angiovue System (software ReVue XR version 2017.1.0.151, Optovue Inc., Fremont, CA, USA) were performed following a standardized protocol based on the split-spectrum amplitude de-correlation algorithm (SSADA), as previously described [37, 38].

The retinal and choriocapillaris (CC) capillary plexus was visualized performing a 6 mm × 6 mm scan over the whole macular region and the percentage area occupied by the microvasculature in the analyzed region defined the vessel density (VD) [39]. The software identified the vessel density of two retinal vascular networks represented by the superficial capillary plexus (SCP) and deep capillary plexus (DCP) [40].

The Angio Vue disc mode automatically segmented the Radial Peripapillary Capillary (RPC) vessel density analyzing the whole papillary region with an area scan of 4.5 × 4.5 mm. The RPC vessel density was analyzed in the superficial retinal layers and extended from the ILM to the retinal nerve fiber layer posterior boundary [41].

From the analysis were excluded the images with a signal strength index less than 40 and residual motion artefacts.

### Statistical analysis

Statistical analysis was performed with the Statistical Package for Social Sciences (Version 28.0 for Windows; SPSS Inc, Chicago, Ill, USA). Data were expressed as mean ± SD, unless otherwise specified. The comparison between patients and controls for OCTA and SD-OCT parameters was performed by non-parametric Mann-Whitney test. The comparison of prevalence between the two groups was made by chi-square test corrected by Fisher’s exact test when necessary. The correlation between hormonal, metabolic, radiological and ophthalmologic parameters was performed by calculating Spearman’s correlation coefficient. Regression analysis and linear mixed models were used to investigate the best predictor of ophthalmologic findings among hormonal and metabolic parameters. In linear mixed models, a random intercept for study partecipants was included to account for the within-subject correlation between measurements from two eyes.

Significance was set at 5%.

## Results

Complete ophthalmologic findings are shown in Table 2. Age (*p* = 0.712) and gender distribution (*p* = 0.413) were similar in patients and controls.

**Table 2:** Results of ophthalmological evaluation

	Patients	Controls	*p*- value
Visual field parameters			
*MD*	−0.96 ± 1.07	−0.42 ± 1.20	0.18
*PSD*	2.27 ± 1.00	2.19 ± 0.45	0.32
OCT parameters (µm)			
GCC	97.21 ± 5.12	103.58 ± 6.01	0.001
*RNFL*	101.5 ± 7.63	108.5 ± 6.81	**0.007**
OCTA parameters (%)			
*SCP*	48.60 ± 3.6	53.53 ± 2.58	**< 0.001**
*DCP*	51.37 ± 6.25	55.56 ± 4.85	**0.009**
*CC*	75.18 ± 2.29	73.12 ± 3.51	**0.05**
*RPC*	49.9 ± 3.74	52.66 ± 3.33	**0.03**

*MD* mean deviation; *PSD* pattern standard deviation; *OCTA* Optical Coherence Tomography Angiography; *SCP* Superficial Capillary Plexus; *DCP* Deep Capillary Plexus; *CC* choriocapillaris; *RPC* Radial Peripapillary Capillary; *OCT* Optical Coherence Tomography; *GCC* Ganglion Cell Complex; *RNFL*: Retinal Nerve Fiber Layer*GCC* Ganglion Cell Complex; *RNFL* Retinal Nerve Fiber Layer; *VD* vessel density; *SCP* Superficial Capillary Plexus; *DCP* Deep Capillary Plexus; *CC* coriocapillar; *RPC*: Radial Peripapillary Capillary*GCC*: Ganglion Cell Complex; *RNFL*: Retinal Nerve Fiber Layer

Mean diagnostic delay before acromegaly diagnosis was 8.5 ± 3.4 years, whereas mean GH and IGF-I levels at acromegaly diagnosis were 15.8 ± 20.9 ng/ml and 813.5 ± 258.4 ng/ml, respectively. Mean adenoma volume was 2162 ± 1930 ml (range 41.6–5765.76 ml). Among patients, 5 out of 12 (41.7%) had arterial hypertension and 6 (50%) had glucose impairment, particularly 2 (16.7%) diabetes mellitus (DM), 2 (16.7%) impaired fasting glucose (IFG), and 2 (16.7%) impaired glucose tolerance (IGT). Hypercholesterolemia and hypertriglyceridemia were present in 5 (41.7%) and 4 (33.3%) patients, respectively.

Visual field parameters (MD, *p* = 0,177, and PSD, *p* = 0,317) were similar between patients and controls.

### Spectral domain optical coherence tomography (SD-OCT)

**Fig. 1:**
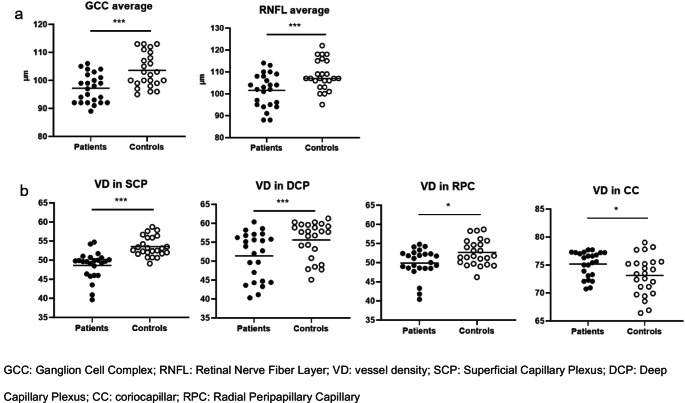
Results of ophthalmological evaluation: SD-OCT (**a**) and OCTA (**b**)

**Fig. 2:**
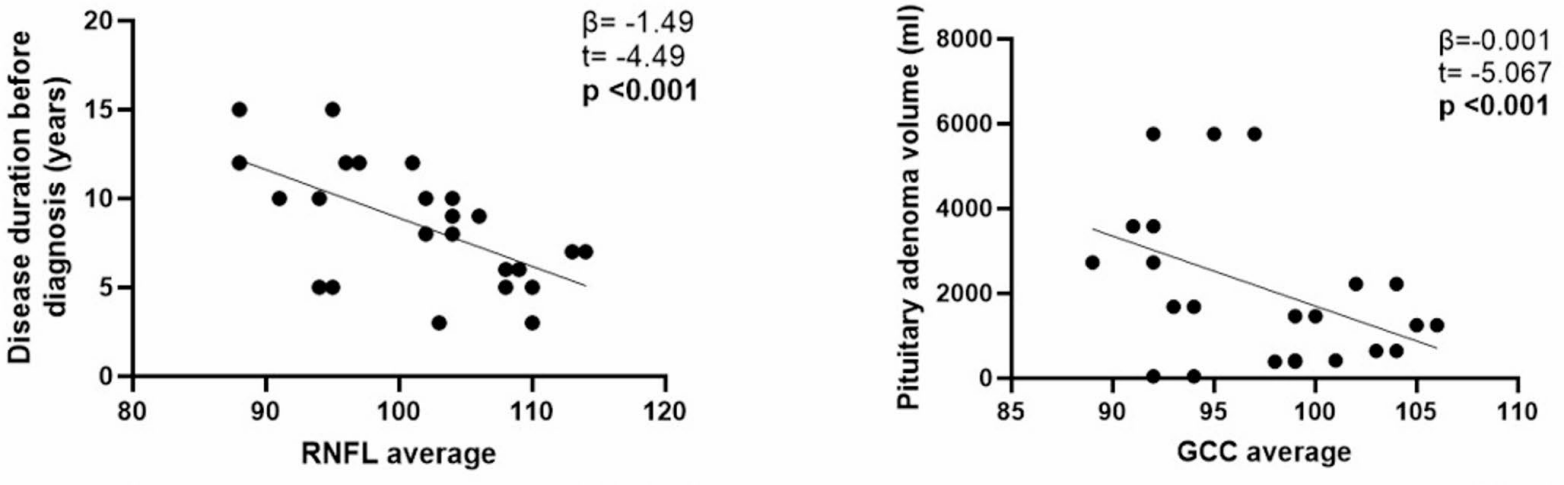
Results of regression analysis concerning SD-OCT parameters

GCC average (*p* = 0.001) and RNFL average (*p* = 0.007) were significantly lower in patients than in controls (Fig. 1a). Patients with diagnostic delay lenght above the median (8.5 years) showed significantly lower GCC (*p* = 0.03) and RNFL (*p* = 0.006) average as compared to those with a shorter diagnostic delay. At correlation study, RNFL was inversely correlated to patients’ age at diagnosis (*r*= −0.521, *p* = 0.009) and diagnostic delay (*r*= −0.583, *p* = 0.003). Moreover, patients with IGF-I levels above the median (897.35 ng/ml) showed slightly higher RNFL average (*p* = 0.08) as compared to those below, resulting RNFL significantly correlated to IGF-I levels at diagnosis (*r* = 0.503, *p* = 0.01). Linear mixed models confirmed diagnostic delay to be the best predictor (β= −1.49, SE = 0.33, t= −4.49, *p* < 0.001) of RNFL average (Fig. 2).

**Fig. 3:**
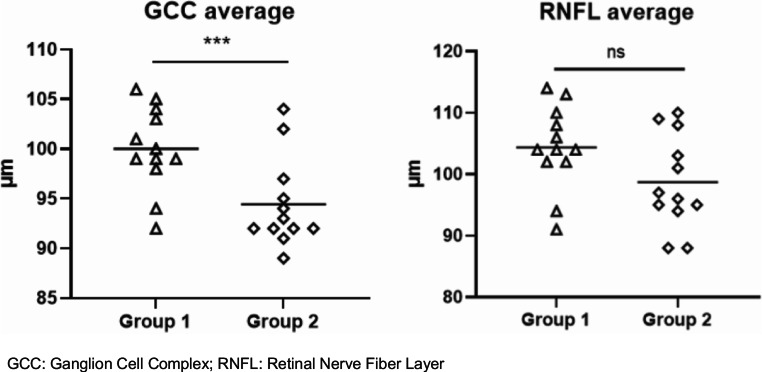
Differences in SD-OCT parameters between patients with pituitary tumor volume below (Group 1) and above (Group 2) the median

According to adenoma volume, patients were divided in two groups: below (Group 1) and above (Group 2) the median (1575 ml). The patients of Group 2 exhibited significantly lower values of GCC average (*p* = 0.008) and slightly lower of RNFL average (*p* = 0.08) despite no impingement of the optic chiasm (Fig. 3), being adenoma volume inversely correlated with GCC average (*r*= −0.423, *p* = 0.04).

Considering acromegaly metabolic comorbidities, patients with dyslipidemia due to hypercholesterolemia and hypetriglyceridemia exhibited significantly lower GCC (*p* = 0.02) and RNFL (*p* = 0.04) average as compared to those without, whereas no significant impact of hypertension and DM were found in SD-OCT parameters.

Linear mixed models confirmed adenoma volume to be the best predictor of GCC average (β=−0.001, SE = 0.0, t=−5.067, *p* < 0.001) (Fig. 2).

### Optical coherence tomography angiography (OCTA)

VD in SCP (*p* < 0.001), DCP (*p* = 0.009), and RPC (*p* = 0.03) was significantly lower in patients as compared to controls, whereas the VD in choriocapillaris (CC, *p* = 0.05) was significantly higher in patients than controls (Fig. 1b). VD in SCP was slightly higher in patients of Group 1 as compared to those of Group 2 (*p* = 0.06), resulting the VD in SCP inversely correlated with adenoma volume (*r*= −0.425, *p* = 0.04). Only among patients of Group 2, VD in RPC inversely correlated with GH (*r*= −0.794, *p* = 0.002) and IGF-I levels (*r*= −0.624, *p* = 0.03). Among patients with acromegaly, VD in RPC was significantly lower in patients with diagnostic delay greater than 8.5 years (median, *p* = 0.009) and in patients with fasting glucose above 90 mg/dl (median, *p* = 0.04) as compared to those below. Furthermore, patients with hypertension showed a slightly lower VD in RPC as compared to those with normal blood pressure (*p* = 0.09). At correlation study, VD in RPC was inversely correlated to diagnostic delay (*r*= −0.477,*p* = 0.02), fasting glucose (*r*= −0.471, *p* = 0.02), and triglycerides levels (*r*= −0.389, *p* = 0.06). Linear mixed models confirmed triglycerides to be the best predictor of VD in RPC at the regression analysis (β=−0.199, SE = 0.074, t=−2.700, *p* = 0.02). Similarly, a trend toward significance was found between VD in DCP and HbA1c levels (*r*= −0.375, *p* = 0.07). Patients with metabolic comorbidities at diagnosis exhibited a significantly lower VD in SCP (*p* = 0.04) as compared to those without metabolic comordibidities.

OCTA parameters significantly correlated with SD-OCT parameters only among acromegaly patients. Particularly, a significant correlation was found between the VD of the SCP and DCP (*r* = 0.462,*p* = 0.02), RPC (*r* = 0,406, *p* = 0.05), GCC (*r* = 0.458, *p* = 0.02), as well as between the VD of the RPC and CC (*r* = 0.452, *p* = 0.03), GCC (*r* = 0.451, *p* = 0.03), RNFL (*r* = 0.384, *p* = 0.06). Furthermore, GCC and RNFL were significantly correlated (*r* = 0.675, *p* < 0.001).

No significant correlation between OCTA and SD-OCT parameters was found in the control group.

## Discussion

The present prospective study has first investigated nervous retinal structure by using SD-OCT and microvasculature of the retina and the choroid by using OCTA in newly diagnosed treatment-naïve acromegaly patients without optic chiasm compression due to the pituitary adenoma. So far, available data about retina and choroid are conflicting and collected in heterogeneous populations including patients already treated with neurosurgery and/or SRLs. This is an important limitation since both surgical and medical treatments might positively influence nervous and vascular structures (8, 29).

The interest for retinal impairment in acromegaly has raised from evidence in preclinical studies that GH and IGF-I receptors are expressed at this level. GH is expressed in the human retina and vitreous fluid, and might play a role in the pathophysiology of some ophthalmic disorders [8, 42], either GH receptors are expressed in the cornea, choroid coat, and retina [8], suggesting the existance of an autocrine and/or paracrine regulation of GH effects in the eye. In animal models of acute retinal injury, long-term treatment with GH for 21 days has been shown to facilitate the early retinal proliferative response and to increase regeneration of the inner plexiform layer and the inner nuclear layer, leading to a general improvement of the retinal structure [43]. These effects are also enhanced by the upregulation of several genes involved in retinal regenerative pathways following long-term chronic GH treatment [44]. Similarly, IGF-I and IGF-I receptors are expressed in the mammalian retina [40], and in primary cultures the treatment with IGF-1 has been demonstrated to determine mild, generalized, and reversible retinal oedema [45]. Concerning clinical studies, GH deficiency has been found to be associated with optic nerve hypoplasia and reduced retinal vascularity [12, 13], whereas opthalmologic manifestations of acromegaly may include increased corneal thickness, melanocytic adenomas of the choroid, retinal pigmentary changes, thickening of the extraocular muscles, proptosis, and ophthalmoplegia [4–7].

Previous studies [15–19] evaluating retinal structure by using SD-OCT in acromegaly patients reported inconclusive results. Indeed, two studies reported that acromegaly may induce RNFL thinning [17, 18], whereas other studies found a similar RNFL thickness between patients and controls [15, 16, 19, 20]. Of note, these patients were already treated for acromegaly with surgery or medical therapy, therefore it cannot be excluded that the finding of no difference between acromegaly patients and controls in these studies could be ascribed to a possible improvement in ophthalmologic structures due to the biochemical and tumoral control of acromegaly.Furtheromore, these studies included also patients with optic chiasm compression [15–19], therefore results provided in these investigations might be biased. Only one study [20] evaluated untreated patients with SD-OCT and Enhanced-Depth Imaging OCT (EDI-OCT) to investigate retinal and choroidal thickness, respectively. Compared with healthy controls, these patients exhibited significantly increased foveal retinal, subfoveal choroidal, and Haller’s layer thicknesses, with no differences in Sattler’s/choriocapillaris layer thickness [20]. Otherwise, GCC and RNFL averages were significantly lower in patients than controls in the current study. These conflicting results could be determined by different characteristics of the naïve patients considered in the two studies such as patient’s age and acromegaly duration which have been demonstrated to be inversely correlatod to GCC and RNFL thickness in the current study. Indeed, patients’ age was higher and diagnostic delay longer in patients of the current study as compared to those of the previous study. In this light, further study on larger populations are required to clarify the role and the burden of GH and IGF-I excess on nervous retinal structure.

So far, optic chiasm compression has been reported as the main responsible for RNFL thinning in presence of pituitary adenomas. Particularly, the RNFL thickness has been first investigated in 29 acromegaly patients undergone surgery as compared to 38 healthy controls [18]. Althoug no difference in mean RNLF thickness in superior quadrants was found between patients and controls, the mean RNLF thickness of the inferior quadrant was significantly lower in acromegaly patients with macroadenoma, than patients with microadenoma and healthy controls [18]. Similarly, RNFL [16, 17] and GCC [16] thickness were found to be significantly lower in patients with biochemically controlled acromegaly after surgery and/or medical therapy with SRLs compared to healthy controls. However, these studies failed to demonstrate a correlation between GH and IGF-I levels and RNLF thickness, thus leading to the conclusion that RNLF thinning might been ascribed to the macroadenoma-induced optic chiasm compression, as well as to arterial hypertension, sleep apnea and/or papilloedema rather than to the hormonal status *per se* [15, 17, 18]. On the other hand, foveal and choroidal thickness has been reported to be increased in acromegalic patients treated either with surgery or medical therapy with SRLs monotherapy or in association to cabergoline [15]. In line with these previous findings, in the present study GCC and RNFL averages were significantly lower in patients than in controls. Noteworthy, the present study only included patients without chiasmal compression and all but one patient of the present cohort harbored a pituitary macroadenoma. In our previous study, GCC and RNFL thinning may occur in newly diagnosed patients with functioning and non-functioning pituitary macroadenomas, even in absence of chiasmal compression [21]. Interestingly, the average circumpapillary RNFL was found significantly lower in patients with acromegaly as compared to those with Cushing’s disease, and superior RNFL significantly lower in patients with acromegaly as compared to patients with prolactinoma and Cushing’s disease [21]. Altogether, the current results raise the hypothesis that, besides adenoma size and mass effect, GH and IGF-I excess *per se* might cause retinal ganglion cell dysfunction although the biological mechanisms underlying this effect are currently unknown. It is important to point out that most of the available studies on retinal nerve function concern GH deficiency and the impact of its replacement and not GH-IGF-I excess. As a consequence, the potential mechanisms whereby GH-IGF-I excess lead to ganglion cell dysfunction can be hypothesized and proposed but need to be clarified in further focused studies. Several in vitro studies on cellular lines demonstrated that GH leads to increased retinal ganglion cell survival, through the inhibition of the apoptosis process. Particularly, exogenous chicken GH was also found to similarly increase the expression of neurotrophin 3, which supports neuronal growth and differentiation, guiding the formation of synaptic connections and the formation of neural networks, and promotes neuronal survival by preventing apoptosis [46]. Further, an increased expression of neurotrophin 3 has been found in GH-secreting pituitary adenomas and has been hypothesized to be involved in pituitary tumour growth and progression [47]. Therefore, it could be hypothesized that in acromegaly patients the prolonged exposure to GH leads to an alteration of the balance of growth and apoptosis of retinal ganglion cells, thus promoting abnormal growth, creation of dysfunctional connections and resulting in the alteration of synaptic transmission. Data about the potential role of IGF-I excess on ganglion cell dysfunction are totally lacking and require future investigations. The findings of this study suggest a major role of length of the exposure to GH and IGF-I excess on the damage of nervous structures of the retina, therefore SD-OCT should be performed at diagnosis particularly in patients with a longer acromegaly diagnostic delay duration.

To date, few studies have been conducted using OCTA to evaluate a potential vascular damage in patients with acromegaly [25–28]. However, also these studies investigated after several years from the diagnosis patients who were already treated for acromegaly, mainly with neurosurgery [29]. Decrease in VD in patients compared to controls have been reported in all retinal macular area [25] or only DCP [26] or RPC [28]. IGF-I levels and disease duration have been demonstrated to be inversely correlated to VD in DCP [26]. Consistenly, in the current study VD in SCP, DCP, and RPC have been found to be significantly lower in patients as compared to controls. Further, the negative impact of IGF-I excess and diagnostic delay on retinal microvasculature has been confirmed in this study.

The current is the first study investigating the role of acromegaly metabolic comorbidities on retinal microvasculature, demonstrating that, beyond the direct negative impact of GH and IGF-I excess, also arterial hypertension, impaired glucose metabolism, and high triglycerides levels are capable of getting worse the vascular retinal damage. Indeed, retinopathy is a well-known complication of diabetes mellitus [48], and OCTA currently represents a fundamental tool for its diagnosis and prognosis [48], whereas less is known about the OCTA role as potential tool for the evaluation of retinopathy in patients with arterial hypertension [49]. As acromegaly patients are at high-risk to develop these cardiometabolic complications and could be affected by these complications already at the time of diagnosis, retinal evaluation with OCTA should be of more interest since both acromegaly *per se* and its cardiometabolic complications could cause retinal vascular damage.

Differently from retinal findings, VD in CC resulted significantly higher in patients than controls in this study. In a previous study, both choroidal thickness anf flow have been demonstrated to be higher in acromegaly patients than controls [27]. Difference in retinal and choroidal vascular findings could be attributed to their anatomic differences. Indeed, the vascular structure of the choroid reportedly has high blood flow [50]. Based on this evidence, in acromegaly an increasing choroidal capillary permeability leading to a further greater blood flow and consequently an increase in choroidal thickness might be assumed [27]. However, this hypothesis is still lacking of a definitive confirmation in clinical settings.

OCTA parameters have been found to be correlated to SD-OCT parameters only in acromegaly patients and not in controls. In healthy subjects VD and RNFL thickness have been demonstrated to be directly correlated each other [51], suggesting a connection between an healthy status of both the nervous structure and the vascular structure. Unfortunately, a similar correlation was not found in the current study likely due to the small number of controls.

The strength of this study is to investigate an underrecognized complication of the prolonged exposure to GH/IGF-I excess. Nevertheless, one might argue that 12 patients are too scant to draw definitive conclusions about the impact of active acromegaly on retinal structure and vasculature. However, this is a pilot study and the current results deserve to be confirmed and further explored in future studies on largest cohort. Anyway, from a statistical point of view, twelve acromegaly patients can be considered fully representative of the disease, also considering the known rarity of this condition. As expected, most of the acromegaly patients recruited for the current study had concomitant cardiometabolic complications, known to influence retinal structure and vasculature [48, 49]. Note to worth, it is uncommon to find acromegaly patients without systemic complications such as hypertension, hyperglycemia, and dyslipidemia. Therefore, the additional analysis of these complications as determinants of retinal structure damage in addition to GH and IGF-I excess and tumor volume performed in this study is a further strength of this investigation, allowing to elucidate the role of the whole disease burden on retinal impairment. Notably, the results of the current study have demonstrated that disease severity (i.e. IGF-I and adenoma volume) and diagnostic delay, rather than systemic complications, exert a direct impact on retinal damage. Similarly, also OCTA parameters resulted correlated to disease activity and duration with the exception of VD in RCP, which resulted correlated to TG levels. This is a completely novel finding as no previous article on other patients, such as those with diabetes or hypertension, has ever reported this data on TG. Further, this study demonstrated that OCTA parameters significantly correlated with SD-OCT parameters only among acromegaly patients and not amog controls, thus suggesting that a potential difference in mechanisms of retinal damage between people affected and not affected by acromegaly cannot be excluded.

On the other hand, while a careful analysis of hormonal excess and relative cardiometabolic consequences has been performed in all acromegaly patients, a similar investigation was not feasible in healthy controls, whose metabolic parameters were not available at the time of the study entry, although previous assessments had definitively excluded the diagnoses of diabetes mellitus, hypertension and dyslipidemia.

## Conclusions

As compared to controls, acromegaly patients exhibited an impairment either in nervous and in vascular retinal structure at the time of diagnosis, regardless chiasm compression by pituitary adenoma. Nervous retinal structure seems to be mainly affected by diagnostic delay before diagnosis and pituitary adenoma volume, even in the absence of chiasmatic compression, rather than by a direct effect of GH and IGF-I excess. Conversely, GH and IGF-I seem to be able to induce a direct damage of retinal microvascularization. SD-OCT and OCTA may represent potential tools to early detect retinal damage in acromegaly patients. Further studies on wider cohorts are needed in order to assess the potential reversibility of both nervous and vascular retinal damage with the restoration of normal GH and IGF-I levels and to find out whether surgical and medical treatments could differently affect retinal structure.

## Data Availability

The datasets generated and analysed during the current study are available upon specific request to the corresponding author.
